# Modeling homophily in dynamic networks with application to HIV molecular surveillance

**DOI:** 10.1186/s12879-023-08598-x

**Published:** 2023-10-04

**Authors:** Victor DeGruttola, Masato Nakazawa, Tuo Lin, Jinyuan Liu, Ravi Goyal, Susan Little, Xin Tu, Sanjay Mehta

**Affiliations:** 1 Division of Biostatistics and Bioinformatics Herbert Wertheim School of Public Health and Human Longevity Science, University of California, 9500 Gilman Dr., 92093-0628 San Diego, La Jolla, CA USA; 2 Pacira Biosciences, San Diego, CA USA; 3 Vanderbilt University, Department of Medicine, Nashville, USA; 4 Division of Infectious Diseases and Global Public Health, University of California San Diego, La Jolla, CA USA; 5 Veterans Affairs, San Diego Healthcare System, San Diego, CA USA

**Keywords:** Homophily, Dynamic networks, Viral genetic linkage

## Abstract

**Background:**

Efforts to control the HIV epidemic can benefit from knowledge of the relationships between the characteristics of people who have transmitted HIV and those who became infected by them. Investigation of this relationship is facilitated by the use of HIV genetic linkage analyses, which allows inference about possible transmission events among people with HIV infection. Two persons with HIV (PWH) are considered linked if the genetic distance between their HIV sequences is less than a given threshold, which implies proximity in a transmission network. The tendency of pairs of nodes (in our case PWH) that share (or differ in) certain attributes to be linked is denoted homophily. Below, we describe a novel approach to modeling homophily with application to analyses of HIV viral genetic sequences from clinical series of participants followed in San Diego. Over the 22-year period of follow-up, increases in cluster size results from HIV transmissions to new people from those already in the cluster–either directly or through intermediaries.

**Methods:**

Our analytical approach makes use of a logistic model to describe homophily with regard to demographic, clinical, and behavioral characteristics–that is we investigate whether similarities (or differences) between PWH in these characteristics are associated with their sequences being linked. To investigate the performance of our methods, we conducted on a simulation study for which data sets were generated in a way that reproduced the structure of the observed database.

**Results:**

Our results demonstrated strong positive homophily associated with hispanic ethnicity, and strong negative homophily, with birth year difference. The second result implies that the larger the difference between the age of a newly-infected PWH and the average age for an available cluster, the lower the odds of a newly infected person joining that cluster. We did not observe homophily associated with prior diagnosis of sexually transmitted diseases. Our simulation studies demonstrated the validity of our approach for modeling homophily, by showing that the estimates it produced matched the specified values of the statistical network generating model.

**Conclusions:**

Our novel methods provide a simple and flexible statistical network-based approach for modeling the growth of viral (or other microbial) genetic clusters from linkage to new infections based on genetic distance.

**Supplementary Information:**

The online version contains supplementary material available at 10.1186/s12879-023-08598-x.

## Introduction

Many complex systems, such as disease transmission among individuals in a population, can be represented as networks, in which nodes and edges represent entities in the systems and the connections among them. Systems that evolve over time (e.g., links among entities are formed over time), can be represented as dynamic networks. One common process underlying formation of connections is homophily, i.e. the tendency of nodes in a dynamic network that share features to be linked. Below we describe a novel approach to model homophily and apply this approach to modeling HIV transmissions among persons with HIV (PWH). We represent PWH as nodes in a network, and potential transmissions as links between two nodes. HIV molecular surveillance techniques applied to HIV genetic sequences are used to establish these links. We refer to the resulting network as a viral genetic linkage network. As in previously reported analyses, two PWH are considered linked if the genetic distance between their HIV sequences is less than a given threshold [23, 25]. Previous studies have shown that a short genetic distance between two infected PWH implies proximity in a transmission network–in the sense that either one of the infected people directly transmitted the virus to the other or indirectly transmitted it through a small number of intermediaries [2]. This belief arises from the fact that HIV evolves rapidly and therefore infections with similar genetic sequences are likely linked by recent transmission events [24].

Public health officials can make use of information on how homophily affects growth of viral genetic linkage networks over time. They can do so by providing additional support (such as pre-exposure prophylaxis or PrEP) to currently uninfected individuals who are likely to join fast-growing genetic clusters, i.e. groups of PWH each of whom is genetically linked to at least one other member of the group but not to any PWH outside the group. (Such groups are referred to as components in network science literature).

Analyses investigating homophily are intended to help direct field resources in ways that can best contain the HIV epidemic by reducing disease transmission. The modeling of homophily in networks is directly related to one of the four “pillars” (Diagnose, Protect, Treat, and Respond) outlined in the Ending the HIV Epidemic plan by the Centers for Disease Control and Prevention (CDC); the Respond pillar focuses on identifying and then acting on outbreaks by providing prevention and treatment services [11]. Molecular epidemiology, which relies on genetic linkage described above, is a key tool to identify fast-growing clusters of related transmissions, and to direct responses to these potential outbreaks [6, 23, 29, 32].

A limitation of this approach is that linked infections between PWH can only be observed if both source and recipient partners can be identified. However, public health responses associated with viral genetic linkage analyses are often coupled with HIV partner notification to provide treatment services to persons who may be living with HIV infection and unaware or prevention services to those who are vulnerable to HIV. Therefore, identifying characteristics that may influence newly-infected people to link to existing genetic clusters could help in identifying persons who are at high risk of acquiring HIV in the future–and thereby in guiding provision of biomedical prevention resources, such as pre-exposure prophylaxis (PrEP) to them. Amirkhanian noted that “Network interventions are feasible and powerful for reducing unprotected sex and potentially for increasing HIV testing uptake” [3].

Estimating homophily in transmission dynamics requires identifying the characteristics (e.g., age, race/ethnicity, neighborhood of residence) that tend to link a newly infected individual with specific viral genetic clusters of PWH–either because such characteristics are similar in this collection of people (an example of homophily) or because they are different (an example of heterophily). Both can occur within the population under study. For example, some newly linked young people might tend to link preferentially to clusters of people of their age, whereas others might link preferentially to clusters of older people. This could lead to differences between the age of newly linked individuals and the mean age of the people in the cluster–and hence a bimodal distribution in the ages of people in clusters themselves. We discuss how to model such possibilities below.

Below we describe the use of a logistic model to describe homophily associated with demographic and behavioral characteristics in a dynamic HIV transmission network. The goal is to provide information about their impact on the nature of forward transmission by identifying characteristics of individuals who may be infected by PWH in the cluster. Our use of independent logistic regression in this setting is not standard; we formally justify this use in the Supplementary Notes and provide further discussion about this issue in “Results” section. The latter also describes a simulation study that further demonstrates the validity of our inferential procedures. We note that logistic models have previously been used to investigate a question that differ from ours (how to describe homophily) but is also based on molecular epidemiological data: what characteristics of newly diagnosed individuals are associated with their joining (or linking to) existing clusters? see [5, 18, 26, 31]. Li et al. used logistic regression directly to address this question [18]; whereas, Rich et al. make use of a suite of machine-learning models (including main-effects boosted logistic regression) to do so. Billock et al. [5] use logistic regression to address another issue of relevance for public health interventions–predicting which clusters will grow–from routine HIV surveillance data in North Carolina. Similarly, Wertheim et al. investigated the possibility of identifying individuals or clusters of individuals most likely to give rise to future HIV cases [31]; and Denis et al. used phylogenetic analyses to investigate the role of unsuppressed infection on linkage to new cases [10].

Our study complements the studies described above; we do not model growth of clusters or predict which newly diagnosed PWH will join a cluster. Instead, our analyses conditions on the fact that a linkage has taken place to address the question of what combination of factors–associated with *both* the newly linked case and the specific cluster it joins–are associated with the event of linkage. This analysis addresses an important gap in the literature: how to enable public health departments to target characteristics of people likely to become infected by those in growing clusters-without the need for individual contact tracing. We also demonstrate that this assessment of homophily can be accomplished using straightforward logistic regression analyses; the underlying mathematical justification requires more sophisticated mathematics, but implementation of the method does not.

## Background

An example of identification of homophily in an HIV transmission network arose in the demonstration of greater viral genetic linkage among Black PWH who have the same income levels compared to those with different income levels [16]. In addition, vulnerability to HIV infection among Black men who have sex with men has been observed to increase when individuals enter high-risk sexual networks characterized by high density and racial homogeneity [3, 14]; such behavioral dynamics might be expected to result in homophily in transmission networks. Similarly among persons who inject drugs (PWIDs), homophily by ethnicity [1] and injecting behaviors [33] has been observed.

Our statistical approach makes use of a logistic model for the analysis of homophily. This flexible model allows for consideration of the extent to which similarities and differences in characteristics (demographic, behavioral, biological) are associated with viral genetic linkage between HIV genetic sequences. The use of a logistic model has connections to a widely used family of network models: exponential random graph models (ERGMs) and their dynamic counter parts, separable temporal ERGMs (STERGMs) [17, 27]. For both of these models, the probability of a link between two nodes can be written as a logistic model [13, 17]. Hunter et al. [13] demonstrates this connection by formulating the probability of a link between nodes *i* and *j*–denoted as $P(Y_{ij} = 1 \vert y_{ij}^c)$ where $y_{ij}^c$ is the rest of the network–using network change statistics. They show that:

1 $$\begin{aligned} logit[P(Y_{ij} = 1 \vert y_{ij}^c)] = \theta ^T*\delta _{g}(y)_{ij}, \end{aligned}$$

where $\theta$ is the vector of model coefficients, *g*(*y*) is a vector of network statistics, and $\delta _{g}(y)_{ij}$ is the change in the value of the network statistic *g*(*y*) that would occur if $y_{ij}$ were changed from 0 to 1 while leaving all of the rest of *y* fixed. Network statistics are statistics that describe properties of networks, such as number of links, degree distribution, or number of triads (sets of 3 nodes all of which are linked to the others).

Although ERGMs provide a flexible approach, they were developed to model social networks [27]. and have limitations in modeling the growth of HIV genetic clusters. In our setting, a newly infected individual links to a single cluster based on their characteristics and that of the cluster. To the authors’ knowledge, this paradigm of a single individual linking to a group can not be modeled with an ERGM. Furthermore, as viral samples from newly infected individuals are sequenced over time, the network size grows. Parameter estimates of ERGMs, however, are valid only for a fixed population size [28]. A similarity between our approach and the formulation of ERGMs is the role played by logistic models, but our use of such models is developed and justified in a different way (see Supplementary Notes). These methods provide a statistical network approach for modeling the growth of viral (or other microbial) genetic clusters through linkage to new infections based on genetic distance.

Clusters of HIV infection tend to grow at highly variable rates [25]. Associations between characteristics of newly linked individuals and HIV viral genetic clusters may strengthen, stabilize, or weaken over time. Our logistic model can accommodate such phenomena by treating the relevant parameters as time varying, though we do not investigate this possibility in our illustrative example.

## Study population

Between July 1, 1996 and March 31, 2018, ART-naïve adult and adolescent ($\mathrm {\ge }$16 year-olds) PWH were prospectively recruited to an observational research study referred to as the University of California San Diego Primary Infection Resource Consortium (PIRC). For details, see Little et al. [20]. Data collected at the baseline visit included: HIV genotype (partial *pol *sequence), testing for bacterial sexually transmitted infections (STIs) (gonorrhea [GC], chlamydia [CT], and syphilis), and routine labs needed for clinical care. Baseline participant characteristics are presented in Table 1.

**Table 1:** Baseline Participant Characteristics for San Diego PIRC Cohort

Number of Participants	*N *= 1119
Race/Ethnicity; n (%)	
White (non-Hispanic)	560 (50.0)
Black (non-Hispanic)	89 (8.0)
Hispanic	341 (30.5)
Other/Unknown	129 (11.5)
Birth Year; median (IQR)	1973 (1965,1982)
Gonorrhea; n(%)^1^	49 (6.6)
Chlamydia; n(%)^1^	62 (8.4)
Syphilis; n(%)^1^	28 (3.8)
Clustered; n(%)^1^	532 (47.5)

Gonorrhea, Chlamydia and Syphilis were not assessed for 377, 378, and 374 participants, respectively

## HIV viral genetic linkage network construction

Population HIV partial *pol* nucleotide consensus sequences were derived for PIRC participants (GenoSure${\circledR }$ MG, LabCorp Specialty Testing Group, South San Francisco or Viroseq v.2.0; Celera Diagnostics, Alameda, CA). If more than one HIV sequence was available for a participant, only the earliest was included in this analysis. We inferred the HIV network by computing all pairwise genetic distances between partial *pol* sequences from each participant (i.e., network node) and connected nodes for which the corresponding genetic distance was less than 1.5% using HIV-TRACE [23]. For further details and information about accessing sequences, see Little et al. [19, 20]. As participants were infected, diagnosed, and sequenced over time, the HIV viral genetic linkage network is dynamic. The network changes at each time a PIRC individual is sequenced for the first time. We denote the PIRC participants with sequences as $\{PWH_{1},\ldots ,PWH_{n}\}$ and the time of sequence collection for participant $PWH_{i}$ as ${t}_{i}$. Note, these times correspond to when individuals are added to the viral genetic linkage network. To create the initial HIV dynamic network, Little et al. [20] identified PWH–i.e., nodes–that did not link to any earlier nodes in the network, which began in 1996. These were defined as “seeds” and followed over time. For each seed or cluster that arose from a seed, we counted the number of incident nodes that subsequently linked to that seed or cluster.

## Network homophily analysis

Little et al. [20] used the network described above to investigate factors associated with growth of clusters through their linkage to people newly infected with HIV (Newly Linked Cases or NLCs), where linkage is defined by degree of genetic similarity between the sequence of virus from the NLC and sequences from members of the cluster. This analysis informs us about characteristics of clusters that are most likely to grow, but provides no information regarding the features of the NLC and of the members of a given cluster (considered jointly) that increase the probability that the NLC will link to that cluster–which is our goal in this section.

In following each seed–or cluster that grew from a seed, we model the linkage event process over time *t* . For each $PWH_{i}$, we have a set of $k_{i}$ clusters available for joining at time $t_{i}$. Let $\textbf{x}_{j}^{i}$ denote a $p\times 1$ vector of cluster-level covariates that characterize the $j^{th}$ cluster that is available at time $t_{i}$ $\left( 1\le j\le k_{i}\right)$. As we describe below, individual-level covariates can include factors like demographics, clinical characteristics (that may change over time), and behavioral characteristics. Cluster-level covariates are summaries of these covariates over the members of a cluster.

For a cluster seed (cluster of size 1), $\textbf{x}_{j}^{i}$ is the covariate for $PWH_{i}$, who defines the $j^{th}$ cluster. For a cluster of size larger than 1, $\textbf{x}_{j}^{i}$ represents a function of the covariates for all of the *PWH* within cluster *j*. Each newly infected ${PWH}_{i}$ either joins one of the clusters available at that time $t_{i}$ or forms its own cluster. In the former case, the number of clusters at time $t_{i}$, is unchanged, but one of the clusters will have a new member, which we denote the $i^{th}$ newly linked case ($NLC^{i}$). The covariate for $NLC^{i}$, denoted by $\textbf{x}_{NLC}^{i}$, will be incorporated into the covariate for the cluster that was joined.

Given $\left\{ \textbf{x}_{1}^{i},\textbf{x}_{2}^{i},...,\textbf{x}_{k_{i}}^{i}\right\}$ and $\textbf{x}_{NLC}^{i}$, we let $m_{i}=k_{i}+1$. Consider a $m_{i}$-dimensional random vector $\textbf{z}^{i}=\left( z_{1}^{i},z_{2}^{i},\ldots ,z_{\left( m_{i}-1\right) }^{i},z_{\left( m_{i}\right) }^{i}\right) ^{\top }$, where $z_{l}^{i}$ is a binary indicator and $\sum _{l=1}^{m_{i}}z_{l}^{i}=1$, i.e., $NLC^{i}$ joined a cluster. Let

$$\begin{aligned} \left\{ d_{1}^{i},d_{2}^{i},...,d_{\left( m_{i}-2\right) }^{i},d_{\left( m_{i}-1\right) }^{i}\right\} =\left\{ d\left( \textbf{x}_{1}^{i},\textbf{x}_{NLC}^{i}\right) ,d\left( \textbf{x}_{2}^{i},\textbf{x}_{NLC}^{i}\right) ,...,d\left( \textbf{x}_{k_{i}-1}^{i},\textbf{x}_{NLC}^{i}\right) ,d\left( \textbf{x}_{k_{i}}^{i},\textbf{x}_{NLC}^{i}\right) \right\} , \end{aligned}$$

where $d\left( \mathbf {\cdot ,\cdot }\right)$ is a function (scalar or vector-valued)–specified by the investigator– of the cluster covariate and the covariate for the NLC. Our interest lies in assessing the degree to which the value of this function–which we denote the homophily covariate–impacts the probability that $\textbf{x}_{NLC}^{i}$ joins cluster *j*, $\left( 1\le j\le k_{i}\right)$.

We fit $k_{i}$ independent logistic regression models as follows:

$$\begin{aligned} z_{j}^{i}\mid d_{j}^{i}\sim \text {Bern}\left( \xi _{j}^{i}\right) ,\quad \xi _{j}^{i}=\frac{\exp \left( \gamma _{0}+\varvec{\gamma }_{1}^{\top }d_{j}^{i}\right) }{1+\exp \left( \gamma _{0}+\varvec{\gamma }_{1}^{\top }d_{j}^{i}\right) },\quad 1\le j\ \le k_{i},\quad \sum \limits _{j=1}^{k_{i}}z_{j}^{i}=1,\quad 1\le i\le n. \end{aligned}$$

where *n* is the total NLCs during the period $1996\le t_i\le 2018$.

The log-likelihood function is given by:

$$\begin{aligned} l=\sum \limits _{i=1}^{n}I\left( \sum \limits _{j=1}^{k_{i}}z_{j}^{i}=1\right) l_{i}=\sum \limits _{i=1}^{n}I\left( \sum \limits _{j=1}^{k_{i}}z_{j}^{i}=1\right) \sum \limits _{j=1}^{k_{i}}z_{j}^{i}\left( 1-z_{j}^{i}\right) \log \left( \xi _{j}^{i}\right) \log \left( 1-\xi _{j}^{i}\right) , \end{aligned}$$

where the indicator $I\left( \sum _{j=1}^{k_{i}}z_{j}^{i}=1\right)$ ensures that the log-likelihood only includes the events in which the $NLC_{i}$ joins a cluster at time $t_{i}$. In the Supplement Note, we show that our approach is equivalent to modeling the linkage event process as a series of multinomial models (in which the dimension of the multinomial vector grows with $k_{i}$ over time) conditioning on $I(\cdot )=1$. This result justifies our approach, which is simpler to implement.

**Fig. 1:**
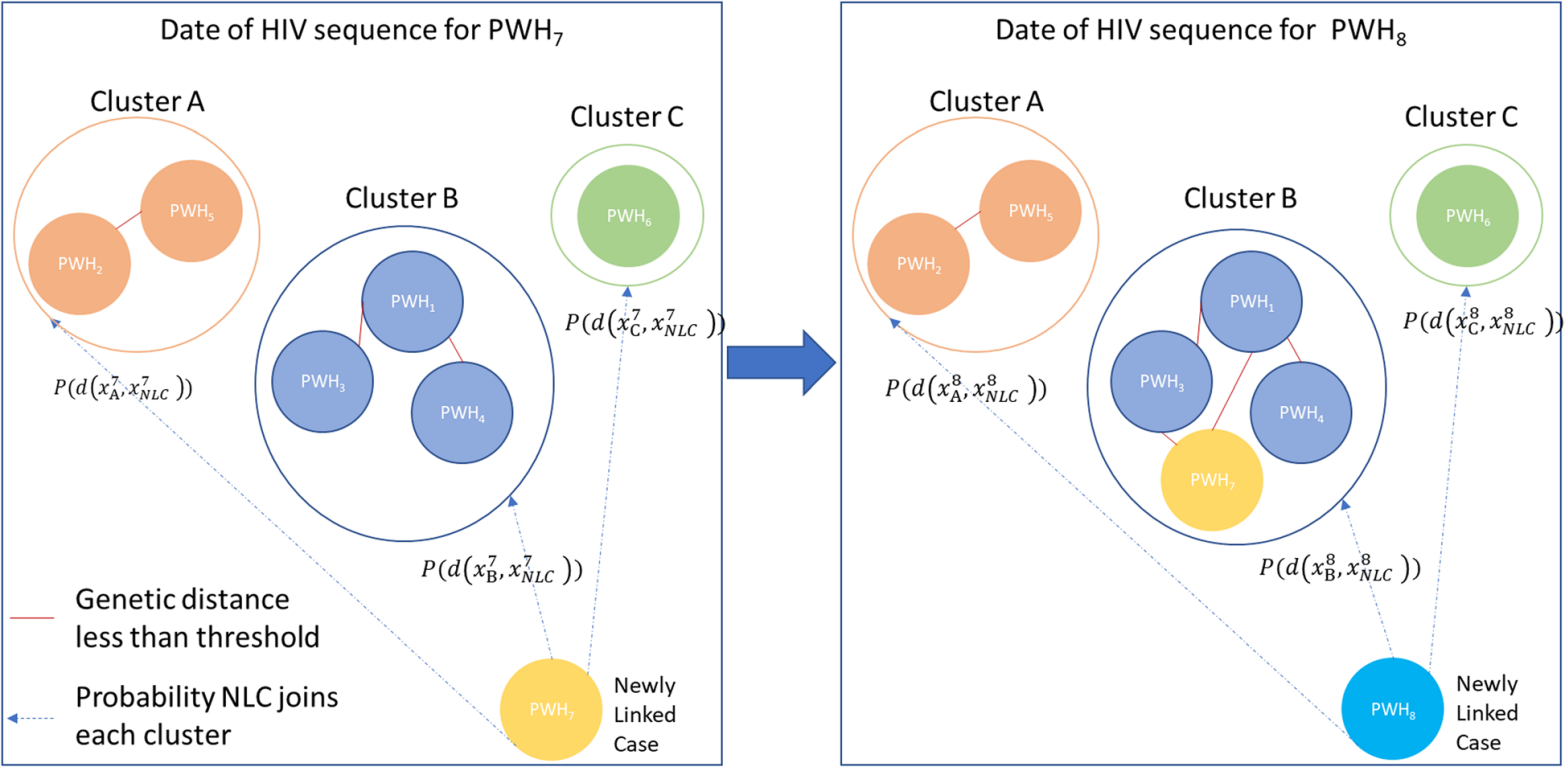
A schematic of the process whereby newly sequenced persons with HIV (PWH) join clusters or else form the seeds of new ones. In the left panel, $PWH_7$ is the newly linked case (NLC); and clusters $C_A$, $C_B$, and $C_C$ are available for $PHW_7$ to join–as indicated by dashed lines. Probabilities that $PHW_7$ will join each one of these clusters are also shown. Solid lines indicate that a the genetic distances between a pair of PWH is below a threshold. The right panel shows that $PWH_7$ linked to Cluster $C_B$; it also displays the same scenario for $PWH_8$ as was displayed for $PWH_7$ in the left panel. Newly sequenced PWH can also form seeds of new clusters, as illustrated by $PWH_6$

Figure 1 presents a schematic of the process by which newly sequenced persons with HIV (PWH) join existing clusters or else form the seeds of new clusters. In the left panel, $PWH_7$ is the newly linked case (NLC); there are 3 clusters $C_A$, $C_B$, and $C_C$ that $PHW_7$ can join–as indicated by the presence of dashed lines. Also displayed are the modeled probabilities that $PHW_7$ joins each one of the clusters, based on the covariates of $PHW_7$ and of the members of that cluster. The solid lines indicate that pairs of PWH have a genetic distance below a threshold. The right panel shows that $PWH_7$ linked to Cluster $C_B$; it also provides a representation of a similar scenario for $PWH_8$ as described above for $PWH_7$. Newly sequenced PW can also form seeds of new clusters, as illustrated by $PWH_6$.

To test the hypothesis that homophily is a driver of the linkage process, we create a homophily covariate (denoted as $d\left( \mathbf {\cdot ,\cdot }\right)$ in the previous section), which characterizes the similarity or difference in characteristics of NLC’s and the clusters available for them to join. For binary outcomes, like Hispanic Ethnicity (HE), we model the homophily covariate as:

$$\begin{aligned} HE_{j,i}=r_{j,i}^{x_{j}^{i}}(1-r_{j,i})^{x_{j}^{i}-1} \end{aligned}$$

where $r_{j,i}$ is the proportion of the members of cluster *j* who are of HE at time ${t_{i}}$; and $x_{l}^{i}=1$ if ${NLC}_{i}$ is positive for HE and 0, if negative. As described above, the NLC is not included in the cluster membership when computing this proportion. We also define a homophily covariate for the absolute value of the difference between the age of the NLC and the average age of the members of clusters *j* at time $t_{i}$ for $j=1,\ 2,\ \dots ,\ k_{i}$. This covariate is calculated as the absolute value of the difference in birth year (${BY}_{i}$) between ${NLC}_{i}$ and the mean for each of the clusters *j* at time $t_{i}$, ($\overline{BY_{j,i}}),\ j=1,\dots ,k_{i}$. Our homophily covariate, denoted birth year difference (BYD), is defined as $BYD_{j,i}=(BY_{i}-\overline{BY_{j,i}}$).

For diseases gonorrhea, chlamydia, and syphilis, STI categorical homophily covariates were created based on Table 2, which defines three categories of homophily: positive, neutral and negative. In this table, *r* is the proportion of cluster members that are STI positive; once again, the NLC was excluded from calculation of *r.* For this analysis, cases with the neutral homophily category were excluded. The reason for this choice is that homophily is harder to interpret in settings when clusters are mixed in STI status.

**Table 2:** Definition of Homophily Status-categorized as Positive, Neutral, and Negative–for STIs. Positive status implies increased probability of linkage; negative, the opposite; neutral implies no effect

Infection Status	Proportion *r* of Cluster members$^{\textrm{a}}$ that are STI positive	Homophily Status
Non-Infected	$0<r<100$	Neutral
Non-Infected	$r=0$	Positive
Non-Infected	$r=100$	Negative
Infected	$0<r<100$	Neutral
Infected	$r=0$	Negative
Infected	$r=100$	Positive

^a^ *r* is the proportion of cluster members (excluding the newly linked case) that are STI positive

Parameter estimation is based on maximum likelihood; and hypothesis tests of the null hypothesis that the homophily covariate has the null value (does not impact risk of joining particular clusters), on the likelihood ratio test. We first consider univariate models to examine whether each predictor was associated with cluster growth and then include those with *p*-value$\mathrm {<}$0.05 in a multivariable model including STI individually and then jointly.

## Results

Baseline participant characteristics are presented in Table 1. Age and ethnicity were available for all participants; but, as indicated in the table, there was a fairly large group of individuals for whom STI information was not available.

### Homophily and sociademographic characteristics

**Fig. 2:**
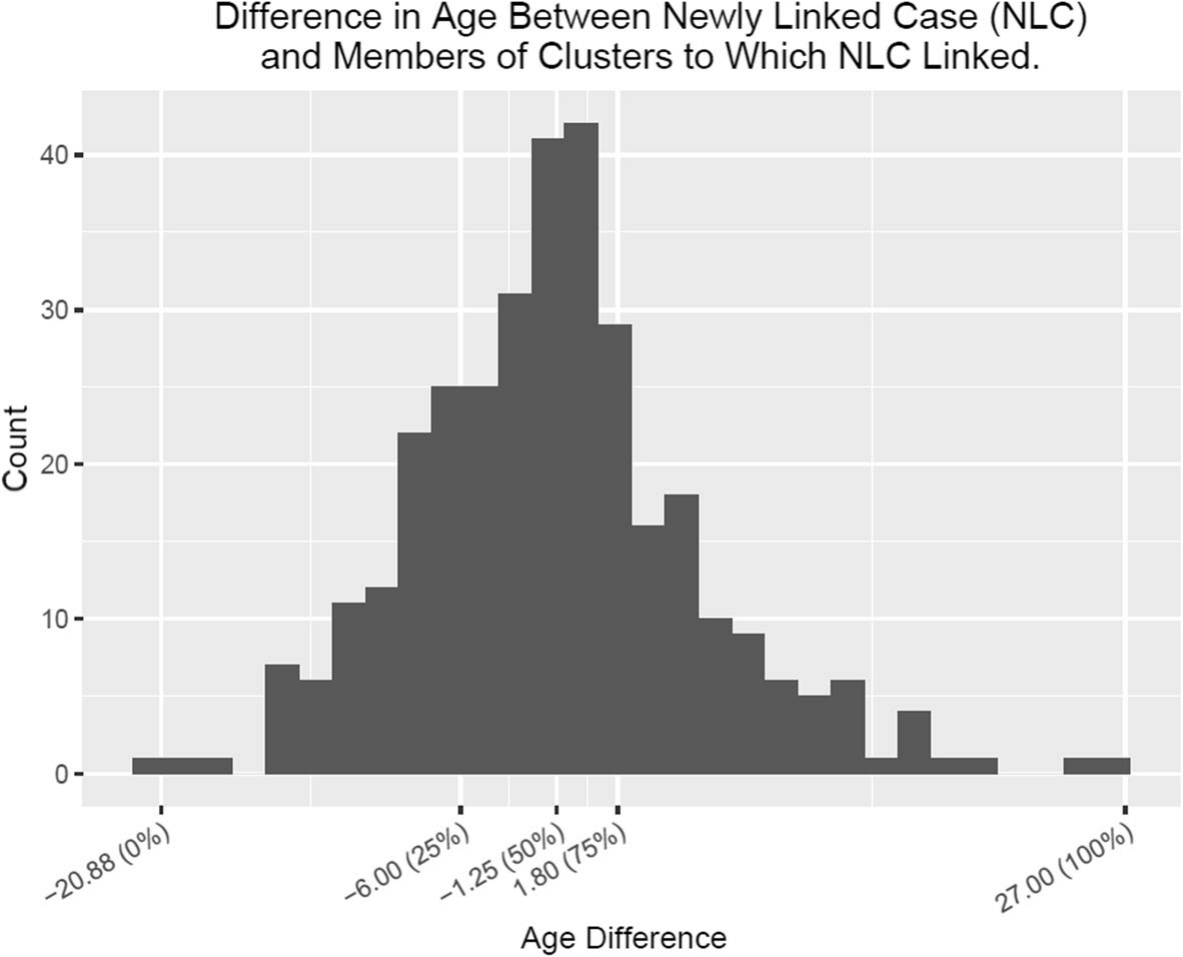
A histogram of the differences in age between the newly linked cases and the clusters to which they were linked

**Fig. 3:**
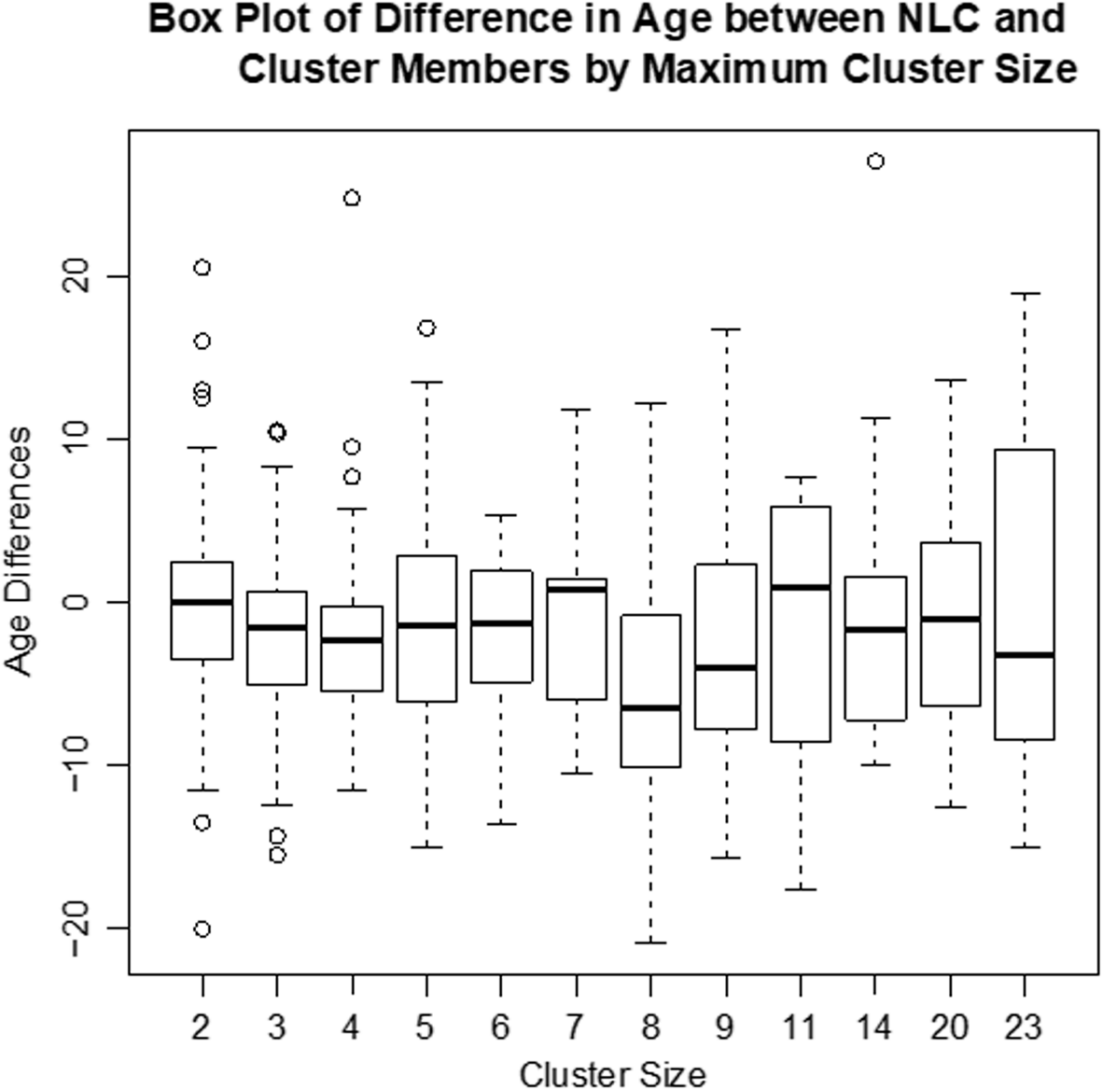
Boxplots for this difference for clusters that achieved different maximum sizes during follow-up

Figure 2 provides a histogram of the differences in age between the newly linked cases and the clusters to which they were linked. The plot shows that the newly linked cases tended to be younger than those in the cluster of linkage; the 25% and 75% percentiles of this distribution are -6.0 and 1.75. We also note that the plot is unimodal. Figure 3 displays the boxplots for this difference for clusters that achieved different maximum sizes during follow-up. No strong relationship between cluster size and this distribution is evident in this figure. In order to accommodate the possibility that linkage could increase with both small and large values of BYD compared to values in the middle range, we can also use functions of it—for example quadratic—in the model in “Network homophily analysis” section.

As shown in Table 3, there was strong positive homophily associated with hispanic ethnicity (HE), and strong negative homophily, with birth year difference (BYD). The second result implies that the larger the difference between the age of the NLC and the average age for an available cluster, the lower the odds of the NLC joining that cluster. In addition, there was a significant interaction between BYD and HE on the odds of linkage. The results from the multivariable model imply that with BYD=0 and when NLC links to a single PWH, the odds of linkage increases by a factor of 3.90 (95% CI 2.86, 5.37) if the NLC and PWH available for linkage share the same HE compared to when they differ. If the NLC links to a cluster of two people of different HE, the odds of linkage is $\sqrt{}3.90$=1.97 compared to when neither share HE with the NLC. The table also shows that for two people of the same HE, for each additional year of difference in BYD, the linkage odds are multiplied by a factor of 0.90 (0.88, 0.93). There was a significant interaction between BYD and HE for negative homophiliy; the odds ratio (95% confidence interval) associated with BYD by HE interaction was 0.93 (0.89, 0.96), p$\mathrm {<}$0.001. This result imples that that for NLC and PWH with the same HE, the odds ratio associated with BYD effect is 0.90 x 0.93= 0.84—which is close to the univariate effect. When we included a quadratic as well as linear effect of BYD along with HE in a model, the quadratic effect was nearly 0 and was associated with a high *p*-value.

**Table 3:** Impact of Hispanic Ethnicity and Birth Year Difference on the Odds of linkage between pairs of sequences. Logistic Model Results with All Cases

	Univariable Models			Multivariable Models		
Effect	OR	OR 95% CI	p	OR	OR 95% CI	p
Abs($\Delta$BY)	0.86	(0.84, 0.87)	$\mathrm {<}$0.001	0.90	(0.88, 0.93)	$\mathrm {<}$0.001
Hispanic	2.44	(1.99, 3.02)	$\mathrm {<}$0.001	3.90	(2.86, 5.37)	$\mathrm {<}$0.001
Abs($\Delta$BY)☓Hispanic				0.93	(0.89, 0.96)	$\mathrm {<}$0.001

The Hosmer Lemershow test for the multivariable model implies a reasonable fit (Chi-square statistic = 8.57, df = 4, *p*-value = 0.073). However, the first two percentile groups—obtained, as is traditional for this test, from the ordered values of estimated probabilities of NLC joining a single PWH or a cluster–had relatively few observed linked events (5 and 37, respectively, Table 4). Collapsing these two categories, yielded a Hosmer Lemershow test that showed stronger support for the model fit (Chi-square statistic = 5.83, df = 3, *p*-value = 0.1204, Table 5, BYD and HE were strongly associated with negative homophiliy and positive homophily respectively. The odds ratio (95% confidence interval) associated with BYD was 0.86 (0.84, 0.87), p$\mathrm {<}$0.001 for both univariate and multivariable models.

**Table 4:** Hosmer Lemershow Test of Goodness of Fit for the Multivariable Model with 6 Bins for the Estimated Probability of Linkage. Similarity of observed and predicted values implies good fit of model to data

P(Y=1)	Observed (Y=0)	Observed (Y=1)	Predicted (Y=0)	Predicted (Y=1)
[7.37e-08,0.000832]	23144	5	23139	10.02
(0.000832,0.00196]	24286	37	24289	33.82
(0.00196,0.00334]	22082	73	22096	58.71
(0.00334,0.00548]	22589	107	22598	97.74
(0.00548,0.0097]	24155	177	24154	177.56
(0.0097,0.0234]	21509	308	21488	329.14

**Table 5:** Hosmer Lemershow Test of Goodness of Fit for the Multivariable Model with 5 Bins (the first two sparse bins were collapsed)

P(Y=1)	Observed (Y=0)	Observed (Y=1)	Predicted (Y=0)	Predicted (Y=1)
[7.37e-08,0.00196]	47430	42	47428	43.85
(0.00196,0.00334]	22082	73	22096	58.71
(0.00334,0.00548]	22589	107	22598	97.74
(0.00548,0.0097]	24155	177	24154	177.56
(0.0097,0.0234]	21509	308	21488	329.14

### Homophily and sexutally transmitted infections

Table 6 shows the frequency distribution of linkages by homophily type (postive, neutral, or negative) and specific sexualy transmitted disease. The upper panel of Table 7 shows that, when investigated individually, none of the STI homophily covariates impacted the probability of linkage. While the current syphillis indicator had a relatively high odds ratio (1.55), the small number of study participants in this category (28) provided limited power; and the 95% confidence interval did not exclude the null value. An additional homophily covariate was considered: the presence or absence of any STI; once again no significant effect was observed. These effects remained qualitatively the same after adjustment for HE and difference in BYD, although the estimated odds ratio for syphillis is somewhat reduced.

**Table 6:** Frequency distribution of linkages by homophily status and sexually transmitted disease

Infection Status of NLC	Cumulative Cluster Proportion	Categorical Homophily	Chlamydia	Gonorrhea	Syphilis	Any STI
Negative	0$\mathrm {<}$r$\mathrm {<}$100	Neutral	212	218	91	163
Negative	r=0	Positive	404	424	588	391
Negative	r=100	Negative	6	3	1	5
Positive	0$\mathrm {<}$r$\mathrm {<}$100	Neutral	51	34	18	100
Positive	r=0	Negative	36	31	12	49
Positive	r=100	Positive	1	0	0	2

**Table 7:** Univariable and Multivariable Logistic Model Results without Neutral Cases. Abbreviations: OR: odds ratio, p: *p*-value; Abs($\Delta$BY): the absolute value of the birth-year difference, CI: confidence interval, STI: sexually transmitted infection

**Effect**	**OR**	**OR 95% CI**	**p**	**OR**	**OR 95% CI**	**p**
	**Univariable Models**					
Gonorrhea	0.90	(0.66, 1.26)	0.53			
Chlamydia	0.88	(0.63, 1.28)	0.49			
Active Syphilis	1.55	(0.93, 2.83)	0.12			
Any STI	1.11	(0.84, 1.49)	0.49			
	**Multivariable Models**					
	**Chlamydia**			**Gonorrhea**		
STI	0.9	(0.66, 1.27)	0.54	0.87	(0.62, 1.25)	0.42
Abs($\Delta$BY)	0.81	(0.79, 0.83)	$\mathrm {<}$0.001	0.81	(0.79, 0.83)	$\mathrm {<}$0.001
Hispanic	3.29	(2.52, 4.37)	$\mathrm {<}$0.001	3.47	(2.65, 4.60)	$\mathrm {<}$0.001
	**Syphilis**			**Any STI**		
STI	1.27	(0.76, 2.33)	0.4	1.07	(0.81, 1.44)	0.66
Abs($\Delta$BY)	0.83	(0.82, 0.85)	$\mathrm {<}$0.001	0.81	(0.79, 0.83)	$\mathrm {<}$0.001
Hispanic	2.77	(2.20, 3.52)	$\mathrm {<}$0.001	3.28	(2.51, 4.35)	$\mathrm {<}$0.001

### Simulation study

To investigate the performance of our methods based on a simulation study, we generated a collection of data sets in a manner intended to reproduce the structure of the observed database. We found the most natural approach to simulation of data was to assume that the joining of an NLC to one of the clusters available at the time of the sequencing of the NLC follows a between-subject multinomial distribution response model (see Eq. 2 below and Supplementary Notes for details).

We define the multinomial response model Multi$_{b}\left( \varvec{\varvec{\eta }}^{i},1\right)$ as:

2 $$\begin{aligned}{} & {} \textbf{z}^{i} \mid \textbf{d}^{i}\sim \text {Multi}_{b}\left( \varvec{\varvec{\eta }}^{i}\right) ,\\{} & {} \varvec{\varvec{\eta }}^{i} =\left( \eta _{1}^{i},\eta _{2}^{i},\ldots ,\eta _{k_{i}}^{i},\eta _{k_{i}+1}^{i}\right) \nonumber \\{} & {} \eta _{j}^{i} =\frac{\exp \left( \beta _{0}+\beta _{1}d_{j}^{i}\right) }{1+\Sigma _{i}},\quad 1\le j\ \le k_{i},\quad \eta _{k_{i}+1}^{i}=\frac{1}{1+\Sigma _{i}},\nonumber \\{} & {} \Sigma _{i} =\sum \limits _{j=1}^{k_{i}}\exp \left( \beta _{0}+\beta _{1}d_{j}^{i}\right) ,\quad \sum \limits _{j=1}^{k_{i}+1}z_{j}^{i}=1,\quad 1\le {i}\le {n}.\nonumber \end{aligned}$$

Then we maximize the log-likelihood function given by:

$$\begin{aligned} l=\sum \limits _{i=1}^{n}l_{i}=\sum \limits _{t=1}^{n}\sum \limits _{j=1}^{k_{i}}z_{j}^{i}\log \left( \eta _{j}^{i}\right) . \end{aligned}$$

Nonetheless, in our example, the analyses are based on logistic regression, which implicitly assumes a different data-generating model: independent Bernoulli. Because of the difference in parameterization of the data generating and the data analysis models, we cannot evaluate the validity of our approach by comparing the estimated and data generating parameters directly. Two approaches were taken to address this issue: 1) Showing analytically that data generated from the between-subject multinomial response model can be modeled in an equivalent way by independent logistic regression (see the Supplementary Notes), and 2) Demonstrating through simulation that the estimated probabilities of linkage to different clusters derived from multinomial and logistic regression models are very nearly identical. We summarize results briefly below and provide details in the Supplementary Note.

Our simulations starts with 5 clusters, to which additional simulated NLC’s are added at each time step; the total number of clusters after the cluster joining process has reached its conclusion is set to 10. We conducted two simulation studies: Study 1 generated covariate data for the 5 original clusters and for the newly link cases from different normal distributions. Study 2 simulates data for one continuous and one binary covariate, based on the observed distributions of birth year and ethnicity in our data. As detailed in the Supplement Note, we simulated the continuous cluster-level covariates from 10 normal distributions, each with a different mean, and the binary cluster-level covariate from 10 Bernoulli distributions, again, each with different mean. To make the 10 distributions of the simulated cluster-level covariates s similar to those of the study data, the 10 normal means have average of 1973 and the 10 Bernoulli means have average of 35%. The number of Monte Carlo simulations was 500.

Results of these simulation studies clearly demonstrate that the two approaches described above yield equivalent results. As expected the approach using the multinomial distribution yielded estimates that were very close to the true values. The codes used for simulation studies in this paper are available on GitHub (https://github.com/tuolin123/Homophily).

#### Sampling density

Often observational studies lack complete coverage of transmission networks, which has implications for analyses and interpretation of results [22]. For example, Volz et al. considered the issue of sampling fraction for analyses of clusters defined phyogenetically, and note that the extent of observed clustering is most sensitive to the fraction of infections sampled [30]. Although our clustering is based on pairwise genetic distance thresholds, extent of clustering in our analysis would be impacted by sampling as well. Therefore, in Supplementary Notes, we investigate conditions under which our methods yield consistent estimates of homophily–through both simulation and more theoretical considerations. We note that a reduced extent of clustering need not necessarily induce bias in estimates of homophily. For example, for the setting of stratified sampling (random selection of participants within strata, such as those defined by ethnicity), estimates of parameters that describe homophily appear to remain valid. Our simulation results, described in detail in Supplementary Notes, support this claim. Nonetheless, if sampling depends on network features, like links between nodes, then this property does not hold.

## Discussion

Our proposed method allows for longitudinal evaluation of homophily in dynamic networks. Because of the somewhat surprising result that analyses of homophily in dynamic networks can be based simply by fitting independent logistic regression models to data at each time of linkage, these analyses can be performed with simple, commonly available software. No expertise in mathematical statistics or network analyses are required to conduct these analyses–though such expertise was required to validate the procedure and demonstrate connections to existing network models (e.g. Exponential Random Graph Models). The focus of our example is on newly identified cases of HIV infection that genetically link to clusters of HIV infected individuals. The method could apply to any other dynamic network in which ties are created or dissolved over time. To incorporate dissolution of ties we could consider a polytomous logistic regression model in which events of both linkage and dissolution of linkage are modeled. Our approach depends on construction of homophily covariates; as we demonstrate, these can be quite general. Here we analyzed covariates of different types to illustrate the flexibility of the approach.

Knowledge of how characteristics of newly linked cases of HIV infection impact probability of joining clusters with particular characteristics provides useful information about transmission dynamics. The homophily covariates may consider both similarity and dissimilarity in these characteristics—and both types of covariates should be considered. We know that both homophily and heterophily may be present and could be detected through model choices and evaluation of their fit. An example arises when there are some people who preferentially select partners based on similarity of age, and others based on difference in age, see for example [9]. In constructing homophily covariates, knowledge of relevant sociological factors as well as in-depth investigation of patterns within the observed data may be useful. For example, preferences for similarity or for difference in age may be associated with other demographic characteristics.

Our analysis based on data from recently infected PWH demonstrates a very strong effect of homophily with regard to Hispanic Ethnicity (HE). In univariable PWH who share the same ethnicity status have 2.44 times of the odds of being linked compared to those with different HE status. Univariable models also show a strong effect of being in the same or nearly the same birth cohort. In multivariables models, the effect of sharing HE status is even stronger. We also found a significant interaction with HE status and birth year; the birth year effect is even greater among those with the same HE. After investigating a variety of ways to model STI homophily, we failed to detect any significant effect–for each of the 3 STIs individually and for all of them jointly. We note that STI status was determined by history at a baseline survey and was not updated over time, and also that a fairly large amount of STI data was missing.

As noted above, homophily may also provide information about transmission of other infections. For example, SARS-CoV-2 has spread more rapidly in certain neighborhoods and certain ethnic/racial and social groups [7]–which may have resulted from homophily in transmission networks [12]. In Japan, Andalibi et al. [4] showed that viral transmission networks of SARS-CoV-2 demonstrated age homophily, as well as homophily between symptomatic and asymptomatic cases, possibly suggesting a virologic effect on transmission. Groups of people who share characteristics (e.g., vacationed at a ski resort [8]) also may be more likely to transmit to each other–either in single transmission or superspreader events. In another example, sexually transmitted infections (STIs) were shown to have been transmitted at greater rates between partners of similar education status in an analysis of five African cities [15]. These examples highlight how knowledge of homophily and heterophily, such as would be revealed in analyses using our methods, could provide insights about transmission dynamics.

Understanding of transmission dynamics can aid in targeting prevention resources. For example, knowing the features of individuals that make them more likely to join certain clusters, because they share (or are dissimilar in) those features, could help prioritize prevention resources to people in clusters with characteristics that make them most likely to experience future growth from linked incident infections. These characteristics may be defined by clinical, demographic, behavioral, and other factors. Similarly, knowing the features of those most likely to join growing clusters may also help in prioritizing PrEP. Together, the knowledge of the clinical and demographic factors associated with growing clusters and the factors associated with persons linking to those clusters provides a blueprint for how to direct limited prevention resources in the most efficient manner.

Despite its potential to advance knowledge on HIV transmission dynamics, ethical concerns about molecular epidemiological studies have been raised; in particular, Mutenherwa et al. conducted interviews with scientists from diverse backgrounds to explore their perspectives on ethical issues associated with research on viral genetic analyses to reveal transmission dynamics [21]. They found that fear of loss of privacy and disclosure of HIV transmission were among the most cited as key ethical concerns. We note that our homophily analysis would allow public health efforts to focus on characteristics of PWH who link to growing clusters rather than attempt contact tracing of PWH included in such clusters. This may reduce the extent of loss of privacy, but still requires the building of the transmission network itself.

Limitations to our analyses include the incompleteness of coverage of the San Diego HIV transmission network under study. Although our analyses are robust to some forms of partial sampling of the transmission network of interest (e.g. stratified sampling), sampling that depends on network features–such as detection of sequences from PWH that relies on contact tracing–can induce bias. Such dependence of sampling on network features will generally induce bias in any analyses of molecular epidemiological data; and stratified sampling will often induce bias as well–for example by reducing the extent of clustering. But our analyses do not depend on observed cluster sizes serving as unbiased estimates of the underlying truth in the population as a whole.

In summary, we have provided a simple approach (based on logistic models) to analyzing the factors that cause PWH to preferentially join clusters with given characteristics. Such information can help enable public health departments to target characteristics of people likely to become infected by those in growing clusters-without the need for individual contact tracing.

### Supplementary Information

**Additional file 1.**

## Data Availability

The datasets needed to reproduce our analysis or perform other relevant analyses related to the current study are available from the corresponding author on reasonable request. In particular, we will provide the following for each node (person with HIV) included in our database: Time order of sequencing, which also serves as an identifier of each node (1,2,3...); Year of sequencing; Year of Birth; Hispanic ethnicity information; the adjacency matrix based on the viral genetic linkage network The genetic sequences themselves would need to be linked to personal data to be useful; but this information cannot be provided to protect confidentiality of participants. However, the sequence information is unnecessary to replicate our analyses or do other such analyses, because the sequences are only needed to compute the adjacency matrix. Given the adjacency matrix, the sequences play no further role in analyses. Note that the timing of the links between nodes is identifiable from the data to be provided, it is the later of the times of sequencing for each linked pair of nodes.
