# Supplementary material for: Modeling homophily in dynamic networks with application to HIV molecular surveillance
Source: BMC Infect Dis. 2023 Oct 4;23:656. doi: 10.1186/s12879-023-08598-x (PMC10548762; doi:10.1186/s12879-023-08598-x)
Supplement: Supplementary file 1 — Additional file 1. [file 12879_2023_8598_MOESM1_ESM.pdf]

1 Supplementary Note

A Models for Homophily

A.1 Data Generating Process and Simulation

We model growth of clusters over time t $(1 \le t \le \infty)$. For each t, we have a set of k_t clusters at time $t = 1$, with $\mathbf{x}_j^t$ denoting a $p \times 1$ vector of cluster-level variables, i.e., $\mathbf{x}_j^t$ determines the characteristics of the k_t clusters $(1 \le j \le k_t)$. For a cluster of size equal to 1, $\mathbf{x}_j^t$ is the variable for the case defining the cluster. For a cluster of size larger than 1, $\mathbf{x}_j^t$ represents some type of aggregate of all cases within the cluster $(1 \le j \le k_t)$. We have a newly infected case $\mathbf{x}_{NLC}^{t+1}$ at $t+1$, which either one of the clusters at t or form its own cluster, where $\mathbf{x}_{NLC}^{t+1}$ is also a $p \times 1$ vector. In the former case, the number of clusters at time t is still $k_{(t+1)} = k_t$, but one of the clusters, say j, will have a new member and its cluster-level variable $\mathbf{x}_j^{t+1}$ will be a function of $\mathbf{x}_j^t$ and $\mathbf{x}_{NLC}^{t+1}$. In the latter case, the number of clusters at time $(t+1)$ will increase by 1 to $k_{(t+1)} = k_t + 1$, with $\mathbf{x}_{NLC}^{t+1}$ forming the $k_{(t+1)}$th cluster. We assume that $\mathbf{x}_j^t$ [SOMETHING MISSING HERE!!]

We describe the data generating process for these two scenarios below.

Scenario (1) $\mathbf{x}_{NLC}^{t+1}$ joins one of the jth clusters at time t, in which case the number of clusters at time $t+1$ remains the same, $k_{(t+1)} = k_t$, but the jth cluster will have a new cluster-level variable to reflect the addition of $\mathbf{x}_{NLC}^{t+1}$:

$$\left\{\mathbf{x}_1^{t+1}, ..., \mathbf{x}_j^{t+1}, ..., \mathbf{x}_{k_{(t+1)}}^{t+1}\right\} = \left\{\mathbf{x}_1^t, ..., \mathbf{x}_j^{t+1} = \mathbf{h}\left(\mathbf{x}_j^t, \mathbf{x}_{NLC}^{t+1}\right), ..., \mathbf{x}_{k_t}^t\right\},$$

where $\mathbf{h}(\cdot,\cdot)$ is a $p \times 1$ vector-valued function to combine $\mathbf{x}_j^t$ and $\mathbf{x}_{NLC}^{t+1}$ to define $\mathbf{x}_j^{t+1} = \mathbf{h}\left(\mathbf{x}_j^t, \mathbf{x}_{NLC}^{t+1}\right)$.

Scenario (2) $\mathbf{x}_{NLC}^{t+1}$ forms its own cluster at time at time $t+1$, in which case the number of clusters at time $t+1$ will grow by one to $k_{(t+1)} = k_t + 1$ and the $k_{(t+1)}$ clusters at $t+1$ are given by:

$$\left\{\mathbf{x}_1^{t+1}, \mathbf{x}_2^{t+1}, ..., \mathbf{x}_{k_{(t+1)}-1}^{t+1}, \mathbf{x}_{k_{(t+1)}}^{t+1}\right\} = \left\{\mathbf{x}_1^t, ..., \mathbf{x}_j^t ..., \mathbf{x}_{k_t}^t, \mathbf{x}_{NLC}^{t+1}\right\}.$$

We now discuss how the two scenarios are determined using Between-subject Multinomial Response models.

A.2 Data Generating Model

Given $\left\{\mathbf{x}_1^t, \mathbf{x}_2^t, ..., \mathbf{x}_{k_t}^t\right\}$ and $\mathbf{x}_{NLC}^{t+1}$, let $m_{(t+1)} = k_t + 1$ and consider a $m_{(t+1)}$-dimensional random vector $\mathbf{z}^{t+1} = \left(z_1^{t+1}, z_2^{t+1}, \ldots, z_{m_{(t+1)}-1}^{t+1}, z_{m_{(t+1)}}^{t+1}\right)^\top$, where z_l^{t+1} is a binary indicator and $\sum_{l=1}^{m_{(t+1)}} z_l^{t+1} =$

1. Let

$$\left\{d_1^{t+1}, d_2^{t+1}, ..., d_{\left(m_{(t+1)}-2\right)}^{t+1}, d_{\left(m_{(t+1)}-1\right)}^{t+1}\right\} = \left\{d\left(\mathbf{x}_1^t, \mathbf{x}_{NLC}^{t+1}\right), d\left(\mathbf{x}_2^t, \mathbf{x}_{NLC}^{t+1}\right), ..., d\left(\mathbf{x}_{k_t-1}^t, \mathbf{x}_{NLC}^{t+1}\right), d\left(\mathbf{x}_{k_t}^t - \mathbf{x}_{NLC}^{t+1}\right)\right\},$$

where $d(\cdot,\cdot)$ is a scalar similarity/dissimilarity function to determine if $\mathbf{x}_{NLC}^{t+1}$ joins $\mathbf{x}_j^t$ $(1 \le j \le k_t)$

We assume that $\mathbf{z}^{t+1}$ conditional on $\left\{d_1^{t+1}, d_2^{t+1}, ..., d_{\left(m_{(t+1)}-2\right)}^{t+1}, d_{\left(m_{(t+1)}-1\right)}^{t+1}\right\}$ follows a $m_{(t+1)}$-level Between-subject Multinomial Response model $\text{Multi}_b\left(\widetilde{\boldsymbol{\eta}}^{t+1}, 1\right)$ defined as:

$$\mathbf{z}^{t+1} \mid \left\{d_1^{t+1}, d_2^{t+1}, ..., d_{\left(m_{(t+1)}-2\right)}^{t+1}, d_{\left(m_{(t+1)}-1\right)}^{t+1}\right\} \sim \text{Multi}_b\left(\boldsymbol{\eta}^{t+1}, 1\right), \tag{1}$$

$$\boldsymbol{\eta}^{t+1} = \left(\eta_1^{t+1}, \eta_2^{t+1}, \ldots, \eta_{\left(m_{(t+1)}-2\right)}^{t+1}, \eta_{\left(m_{(t+1)}-1\right)}^{t+1}\right)^\top,$$

$$\eta_j^{t+1} = \frac{\exp\left(\beta_0 + \beta_1 d_j^{t+1}\right)}{1 + \Sigma_{t+1}}, \quad 1 \le j \ \le m_{(t+1)} - 1, \quad \eta_{m_{(t+1)}}^{t+1} = \frac{1}{1 + \Sigma_{t+1}},$$

$$\Sigma_{t+1} = \sum_{j=1}^{m_{(t+1)}-1} \exp\left(\beta_0 + \beta_1 d_j^{t+1}\right), \quad \sum_{j=1}^{m_{(t+1)}} z_j^{t+1} = 1.$$

The size of this multinomial is 1, i.e., $\sum_{j=1}^{m_{(t+1)}} z_j^{t+1} = 1$, and $\boldsymbol{\eta}^{t+1}$ denotes the vector of cell probabilities. Thus only one of the components of $\mathbf{z}^{t+1}$ is 1 and the rest is 0.

Scenario (1) This occurs when $z_j^{t+1} = 1$ for some j $\left(1 \le j \le m_{(t+1)} - 1\right)$. In this case, $\mathbf{x}_{NLC}^{t+1}$ joins the jth cluster at time t and the clusters at time $t+1$ remain the same as at time t, except for the jth cluster that will have a new cluster-level variable to reflect the addition of $\mathbf{x}_{NLC}^{t+1}$ to its members. Thus, $k_{(t+1)} = k_t$ and the cluster variables for time $t+1$ are given by:

$$\left\{\mathbf{x}_1^{t+1}, ..., \mathbf{x}_j^{t+1}, ..., \mathbf{x}_{k_{(t+1)}}^{t+1}\right\} = \left\{\mathbf{x}_1^t, ..., \mathbf{h}\left(\mathbf{x}_j^t, \mathbf{x}_{NLC}^{t+1}\right), ..., \mathbf{x}_{k_t}^t\right\}.$$

Scenario (2) This occurs when $z_{m_{(t+1)}}^{t+1} = 1$, in which case $\mathbf{x}_{NLC}^{t+1}$ forms a new cluster. The number of clusters at time $t+1$ will grow by one to $k_t + 1$. Thus, $k_{(t+1)} = k_t + 1$ and the cluster variables for time $t+1$ are given by:

$$\left\{\mathbf{x}_1^{t+1}, .., \mathbf{x}_j^{t+1}, ..., \mathbf{x}_{k_t}^t, \mathbf{x}_{k_{(t+1)}}^{t+1}\right\} = \left\{\mathbf{x}_1^t, ..., \mathbf{x}_j^t ..., \mathbf{x}_{k_t}^t, \mathbf{x}_{NLC}^{t+1}\right\}.$$

We can readily fit the between-subject multinomial response models in (1) to the data generated. Alternatively, we can fit independent logistic regression, as we did in the analysis of the homophily data. We discuss the basis for this alternative approach next.

A.3 Relationship between Independent Bernoulli and Multinomial Distribution

Consider k independent Bernoulli $z_j \sim Bern(\rho_j)$ $(1 \le j \le k)$ and let $\mathbf{z} = (z_1, z_2, \ldots, z_k)^\top$. Then, $\mathbf{z}$ given $\sum_{j=1}^n z_j = 1$ has a multinomial:

$$\mathbf{z}|\sum_{j=1}^{n} z_j = 1 \sim Mult(\mathbf{p},1), \quad \mathbf{p} = (p_1, p_2, \ldots, p_k)^\top,$$

$$p_j = \frac{\rho_j}{\sum_{l=1}^k \rho_l + \sum_{i \neq j}^k \frac{\rho_j(\rho_j - \rho_i)}{1-\rho_j}}, \quad 1 \le j \le k.$$

To show, first consider p_1:

$$\begin{aligned}
p_1 &= \Pr\left(Z_1 = 1, Z_j = 0 \text{ for all } j \neq 1 \mid \sum_{j=1}^{k} Z_j = 1\right) \\
&= \frac{\rho_1 \prod_{j=2}^k (1-\rho_j)}{\sum_{j=1}^k \rho_j \prod_{l \neq j} (1-\rho_l)} \\
&= \frac{\rho_1 \prod_{j=2}^k (1-\rho_j)}{\sum_{j=1}^k \rho_j \prod_{l=2}^k (1-\rho_l) + \sum_{j=2}^k \rho_j \prod_{l \neq 1,j} (1-\rho_l)(\rho_j - \rho_1)} \\
&= \left(\frac{\sum_{j=1}^k \rho_j}{\rho_1} + \frac{\sum_{j=2}^k \rho_j \prod_{l \neq 1,j} (1-\rho_l)(\rho_j - \rho_1)}{\rho_1 \prod_{j=2}^k (1-\rho_j)}\right)^{-1} \\
&= \left(\frac{\sum_{j=1}^k \rho_j}{\rho_1} + \sum_{j=2}^{k} \frac{\rho_j(\rho_j - \rho_1)}{\rho_1(1-\rho_j)}\right)^{-1} \\
&= \frac{\rho_1}{\sum_{j=1}^k \rho_j + \sum_{j=2}^k \frac{\rho_j(\rho_j-\rho_1)}{(1-\rho_j)}}.
\end{aligned}$$

In general, the multinomial event probability p_j is given by:

$$\Pr\left(Z_j = 1, Z_l = 0 \text{ for all } l \neq j \mid \sum_{l=1}^{k} Z_l = 1\right) = \frac{\rho_j}{\sum_{l=1}^n \rho_l + \sum_{l \neq j} \frac{\rho_l(\rho_l - \rho_j)}{1-\rho_l}}.$$

Thus by viewing the data generating process as the conditional distribution of independent Bernonlli's $z_1, z_2, \ldots, z_k^\top$ under the constraint $\sum_{j=1}^n z_j = 1$, we can fit the data generated using independent logistic regression.

A.4 Model Fitting

A.4.1 Model Fitting based on Between-subject Multinomial Response Model

Given n newly infected cases, we have observed data:

$$\mathbf{x}_{NLC}^{t+1}, \quad \left\{\mathbf{x}_1^t, \mathbf{x}_2^t, ..., \mathbf{x}_{k_t}^t\right\}, \quad \mathbf{z}^{t+1} = \left(z_1^{t+1}, z_2^{t+1}, \ldots, z_{k_t}^{t+1}, z_{k_t+1}^{t+1}\right)^\top, \quad 1 \le t \le n. \tag{2}$$

If $\mathbf{x}_{NLC}^{t+1}$ joins a cluster at time t, say, j, then $z_j^{t+1} = 1$ for some j $(1 \le j \le k_t)$. If $\mathbf{x}_{NLC}^{t+1}$ forms its own cluster, then $z_{k_t+1}^{t+1} = 1$.

First, we compute the similarity/dissimilarity variables for the clusters:

$$\left\{d_1^{t+1}, d_2^{t+1}, ..., d_{k_t}^{t+1}\right\} = \left\{d\left(\mathbf{x}_1^t, \mathbf{x}_{NLC}^{t+1}\right), d\left(\mathbf{x}_2^t, \mathbf{x}_{NLC}^{t+1}\right), ..., d\left(\mathbf{x}_{k_t}^t, \mathbf{x}_{NLC}^{t+1}\right)\right\}, \quad 1 \le t \le n. \tag{3}$$

Then, we fit a $(k_t + 1)$-level between-subject multinomial response model $\text{Multi}_b\left(\boldsymbol{\eta}^{t+1}, 1\right)$ using maximum likelihood:

$$\begin{aligned}
&\mathbf{z}^{t+1} \mid \left\{d_1^{t+1}, d_2^{t+1}, ..., d_{k_t}^{t+1}\right\} \sim \text{Multi}_b\left(\boldsymbol{\eta}^{t+1}\right), \qquad (4)\\
&\mathbf{z}^{t+1} = \left(z_1^{t+1}, z_2^{t+1}, \ldots, z_{k_t}^{t+1}, z_{k_t+1}^{t+1}\right)^\top, \quad \boldsymbol{\eta}^{t+1} = \left(\eta_1^{t+1}, \eta_2^{t+1}, \ldots, \eta_{k_t}^{t+1}, \eta_{k_t+1}^{t+1}\right)\\
&\eta_j^{t+1} = \frac{\exp\left(\beta_0 + \beta_1 d_j^{t+1}\right)}{1 + \Sigma_{t+1}}, \quad 1 \le j \ \le k_t, \quad \eta_{k_t+1}^{t+1} = \frac{1}{1 + \Sigma_{t+1}},\\
&\Sigma_{t+1} = \sum_{j=1}^{k_t} \exp\left(\beta_0 + \beta_1 d_j^{t+1}\right), \quad \sum_{j=1}^{k_t+1} z_j^{t+1} = 1, \quad 1 \le t \le n.
\end{aligned}$$

The log-likelihood function is given by:

$$l = \sum_{t=1}^{n} l_t = \sum_{t=1}^{n} \sum_{j=1}^{k_t} z_j^t \log(\eta_j^t).$$

A.4.2 Model Fitting based on Independent Logistic Regression

When fitting the Between-subject Multinomial, we need to have full data that includes both Scenario 1 and Scenario 2. In the homophily paper, we only have data for Scenario 1, i.e., we only have data when a newly infected case

$$withcovariate$$

$\mathbf{x}_{NLC}^{t+1}$ joins a cluster at time t. Thus, we only have a subset of the data in (2) under the constraint $z_j^{t+1} = 1$ for some j $(1 \le j \le k_t)$, or $\sum_{j=1}^{k_t} z_j^{t+1} = 1$ $(1 \le t \le n)$.

Based on the relationship between independent Bernoulli and multinomial distribution (see below), we can model the subset of the observed data $\mathbf{z}^{t+1}$ with $\sum_{j=1}^{k_t} z_j^{t+1} = 1$ using $\sum_{t=1}^{n} k_t I\left(\sum_{j=1}^{k_t} z_j^{t+1} = 1\right)$ independent logistic regression models. Thus, given

$$\mathbf{x}_{NLC}^{t+1}, \quad \left\{\mathbf{x}_1^t, \mathbf{x}_2^t, ..., \mathbf{x}_{k_t}^t\right\}, \quad \mathbf{z}^{t+1} = \left(z_1^{t+1}, z_2^{t+1}, \ldots, z_{k_t}^{t+1}\right)^\top, \quad \sum_{j=1}^{k_t} z_j^{t+1} = 1, \quad 1 \leq t \leq n. \tag{5}$$

we again compute $\left\{d_1^{t+1}, d_2^{t+1}, ..., d_{k_t}^{t+1}\right\}$ in (3). Then we fit k_t independent Bernoulli (logistic regression):

$$z_j^{t+1} \mid d_j^{t+1} \sim \text{Bern}\left(\xi_j^{t+1}\right), \quad \xi_j^{t+1} = \frac{\exp\left(\gamma_0 + \gamma_1 d_j^{t+1}\right)}{1 + \exp\left(\gamma_0 + \gamma_1 d_j^{t+1}\right)}, \quad 1 \leq j \leq k_t, \quad \sum_{j=1}^{k_t} z_j^{t+1} = 1, \quad 1 \leq t \leq n.$$

The log-likelihood function is given by:

$$l = \sum_{t=1}^{n} I\left(\sum_{j=1}^{k_t} z_j^{t+1} = 1\right) l_t = \sum_{t=1}^{n} I\left(\sum_{j=1}^{k_t} z_j^{t+1} = 1\right) \sum_{j=1}^{k_t} z_j^t \left(1 - z_j^t\right) \log\left(\xi_j^t\right) \log\left(1 - \xi_j^t\right),$$

where the indicator $I\left(\sum_{j=1}^{k_t} z_j^{t+1} = 1\right)$ ensures that the log-likelihood only includes the samples from the between-subject multinomial response model when the newly infected case at time $(t+1)$ joins a cluster at time t.

B Simulation Study

B.1 Study One

We use simulated data to examine (1) if data generated from the between-subject multinomial response model in Section A.2 can be equivalently modeled by independent logistic regression as described in Section A.3 and (2) performance of the independent logistic regression when fit data generated from the between-subject multinomial response model.

For (1), we started with 5 clusters and set 10 as the total number of clusters. The cluster-level variables for the beginning 5 clusters, $\{x_1, x_2, ..., x_5\}$, were simulated from 5 different normal distributions:

$$\left\{x_1^1, x_2^1, ..., x_5^1\right\}, \quad x_j^1 \sim N\left(2j - 1, 0.1\right), \quad 1 \leq j \leq 5.$$

To simulate a sample of size n from the between-subject Multinomial response model $\text{Multi}_b\left(\boldsymbol{\eta}^{t+1}, 1\right)$, for $1 \leq t \leq n$ each newly infected case $\mathbf{x}_{NLC}^{t+1}$ was generated from a normal distribution:

$$\mathbf{x}_{NLC}^{t+1} \sim N\left(2r - 1, 1\right), \quad r \sim U_d\{1, 2, ..., k_t\}, \quad 2 \leq t \leq n,$$

where $U_d\{1,2,...,k_t\}$ denotes a discrete uniform distribution with values $1,2,...,k_t$. We use $d_j^{t+1} = d\left(x_j^t, x_{NLC}^{t+1}\right) = \left|x_j^t - x_{NLC}^{t+1}\right|^{-1}$ (truncated at 0.001 and 100) as the similarity/dissimilarity function and $h\left(x_j^t, x_{NLC}^{t+1}\right) = \frac{1}{2}\left(x_j^t + x_{NLC}^{t+1}\right)$ as the function to integrate each x_{NLC}^t with the cluster-level variable x_j^t of the cluster that x_{NLC}^t joins. We set $n = 1,000$ for our simulation. The event probability $\boldsymbol{\eta}^{t+1}$ for the between-subject Multinomial response model for generating the multinomial response $\mathbf{z}^{t+1}$ in (1) is given by:

$$\eta_j^{t+1} = \frac{\exp\left(\beta_0 + \beta_1 d_j^{t+1}\right)}{1 + \Sigma_{t+1}} = \frac{\exp\left(-0.8 + 0.1 d_j^{t+1}\right)}{1 + \Sigma_{t+1}}, \tag{6}$$

$$\eta_{m_{(t+1)}}^{t+1} = \frac{1}{1 + \Sigma_{t+1}}, \quad \Sigma_{t+1} = \sum_{j=1}^{m_{(t+1)}-1} \exp\left(-0.8 + 0.1 d_j^{t+1}\right).$$

To reduce sampling variability as well as to compare estimates from fitting the between-subject multinomial response and independent logistic regression model, we controlled the total number of clusters to 10, i.e., $k_n = 10$, with the fixed sample size n. Thus, a newly infected case x_{NLC}^{t+1} will not form its own cluster for every t when $\sum_{j=1}^{k_t} z_j^{t+1} = 0$ until a certain number of samples are generated from $\text{Multi}_b\left(\boldsymbol{\eta}^{t+1}, 1\right)$ for a given number of clusters. For example, to grow the initial 5 clusters to 10 clusters, we select 6 subsample sizes, $1 < n_5 < n_6 \ldots < n_9 < n_{10}$, such that for $n_{l-1} + 1 \le t \le n_l$ we generate $\mathbf{z}^{t+1}$ from $\text{Multi}_b\left(\boldsymbol{\eta}^{t+1}, 1\right)$ with k_t clusters, where $n_4 = 0$ and $5 \le l \le 10$. For our simulation study, we set $n_5 = 300, n_6 = 500, n_7 = 700, n_8 = 900, n_9 = 1100, n_{10} = 1500$.

We fit both the between-subject Multinomial response model and independent logistic regression as described in Section in (A.3). For the between-subject Multinomial response model, we obtained: $\widehat{\boldsymbol{\beta}} = \left(\widehat{\beta}_0, \widehat{\beta}_1\right)^\top = (-0.7995, 0.0999)^\top$, which were quite close to the respective true values $\beta_0 = -0.8$ and $\beta_1 = 0.1$. For the independent logistic regression, we obtained: $\widehat{\boldsymbol{\gamma}} = (\widehat{\gamma}_0, \widehat{\gamma}_1)^\top = (-2.0401, \widehat{\gamma}_1 = 0.0963)^\top$. Although $\widehat{\boldsymbol{\beta}}$ and $\widehat{\boldsymbol{\gamma}}$ are not directly comparable, we can compare the fitted $\widehat{\boldsymbol{\eta}}^{t+1}$ and $\widehat{\boldsymbol{\xi}}^{t+1}$ based on substituting $\widehat{\boldsymbol{\beta}}$ and $\widehat{\boldsymbol{\gamma}}$ in place of $\boldsymbol{\beta}$ and $\boldsymbol{\gamma}$. Since the independent logistic regression is fit to the subject vector $\mathbf{z}_{sub}^{t+1} = \left(z_1^{t+1}, z_2^{t+1}, \ldots, z_{k_t}^{t+1}\right)^\top$ of $\mathbf{z}^{t+1} = \left(z_1^{t+1}, z_2^{t+1}, \ldots, z_{k_t}^{t+1}, z_{k_t+1}^{t+1}\right)^\top$, we compare $\widehat{\boldsymbol{\xi}}^{t+1} = \left(\widehat{\xi}_1^{t+1}, \widehat{\xi}_2^{t+1}, \ldots, \widehat{\xi}_{k_t}^{t+1}\right)^\top$ with the normalized subvector $\widehat{\boldsymbol{\eta}}_{sub}^{t+1} = \frac{1}{\widehat{s}_{t+1}}\left(\widehat{\eta}_1^{t+1}, \widehat{\eta}_1^{t+1}, \ldots, \widehat{\eta}_{k_t}^{t+1}\right)^\top$, where $\widehat{s}_{t+1} = \sum_{j=1}^{k_t} \widehat{\eta}_j^{t+1}$.

Shown below are the averaged $\widehat{\boldsymbol{\eta}}_{sub}^{t+1}$ and $\widehat{\boldsymbol{\xi}}^{t+1}$ over the samples $1 \le t \le n_5$ with 5 clusters, $\overline{\widehat{\boldsymbol{\eta}}}_{sub}^{5}$

and $\overline{\overline{\boldsymbol{\xi}}}_{sub}^5$, and over the samples $n_9 \leq t \leq n_{10}$, $\overline{\overline{\boldsymbol{\eta}}}_{sub}^{10}$ and $\overline{\overline{\boldsymbol{\xi}}}_{sub}^{10}$:

$$\overline{\overline{\boldsymbol{\eta}}}_{sub}^5 = (0.1973, 0.1967, 0.2006, 0.2060, 0.1994)^\top,$$

$$\overline{\overline{\boldsymbol{\xi}}}_{sub}^5 = (0.2001, 0.1977, 0.2008, 0.2037, 0.1977)^\top,$$

$$\overline{\overline{\boldsymbol{\eta}}}_{sub}^{10} = (0.1019, 0.1045, 0.1002, 0.0951, 0.1024, 0.0970, 0.0978, 0.1019, 0.0934, 0.1060)^\top,$$

$$\overline{\overline{\boldsymbol{\xi}}}_{sub}^{10} = (0.1017, 0.1091, 0.1022, 0.0875, 0.1004, 0.1008, 0.1028, 0.0997, 0.0938, 0.1021)^\top.$$

In both cases, the averaged event probabilities from the fitted between-subject Multinomial response and independent logistic regression model were quite close to each other.

To see if estimates of $\widehat{\boldsymbol{\gamma}} = (\widehat{\gamma}_0, \widehat{\gamma}_1)^\top$ in the independent logistic regression converge, we simulated a sample size of 450 and 900 homophily cases, i.e., all simulated 450 (900) newly infected cases that join existing clusters. The two sets of estimates are $(\widehat{\gamma}_0, \widehat{\gamma}_1) = (-2.104, 0.113)$ for 450 and $(\widehat{\gamma}_0, \widehat{\gamma}_1) = (-2.051, 0.105)$ for 900. They are close to each other.

B.2 Study Two

In this simulation study, we simulate data from one continuous and one binary predictor based on the distributions of the observed birthy year and ethnicity. The mean birth year is 1973 and the proportion of Hispanic Ethnicity (HE) is 35%. We again set the total number of clusters to 10. We simulate the continuous cluster-level variable from 10 normal distributions with different means and the binary cluster-level variable from 10 Beroulli distributions with different means. To make the 10 distributions of the cluster-level variables similar to those of the study data, the 10 normal means average to 1973 and the 10 Bernoulli means average to 35%.

We again started with 5 clusters with the continuous cluster-level birth year variable, $\{x_{11}, x_{12}, ..., x_{15}\}$, following the 5 different normal distributions:

$$\left\{x_{11}^1, x_{12}^1, ..., x_{15}^1\right\},$$
$$x_{11}^1 \sim N(1968, 1), x_{21}^1 \sim N(1971, 1), x_{31}^1 \sim N(1975, 1), x_{41}^1 \sim N(1978, 1), x_{51}^1 \sim N(1981, 1).$$

The 5 binary cluster-level HE variable, $\{x_{21}, x_{22}, ..., x_{25}\}$, following the 5 different Bernoulli distributions:

$$\left\{x_{21}^1, x_{22}^1, ..., x_{25}^1\right\}, x_{21}^1 \sim Ber(0.25), x_{22}^1 \sim Ber(0.3), x_{23}^1 \sim Ber(0.35), x_{24}^1 \sim Ber(0.4), x_{25}^1 \sim Ber(0.45).$$

To simulate a sample of size n from the between-subject Multinomial response model $\text{Multi}_b\left(\boldsymbol{\eta}^{t+1}, 1\right)$, for $1 \leq t \leq n$ each newly infected case $\mathbf{x}_{NLC}^{t+1} = \left(x_{NLC1}^{t+1}, x_{NLC2}^{t+1}\right)^\top$ was generated from a normal and a Beroulli distribution as follows:

$$\mathbf{x}_{NLC1}^{t+1} \sim N(V_{1r}, 1), \quad \mathbf{x}_{NLC2}^{t+1} \sim Ber(V_{2r}), \quad r \sim U_d\{1, 2, ..., k_t\}, \quad 2 \leq t \leq n,$$

where $U_d\{1,2,...,k_t\}$ denotes a discrete uniform distribution with values $1,2,...,k_t$, V_{1r} and V_{2r} denotes the r-th row of vector V_1 and V_2, and $V_1 = (1968, 1971, 1975, 1978, 1981, 1955, 1961, 1965, 1985, 1991)$, $V_2 = (0.25, 0.3, 0.35, 0.4, 0.45, 0.1, 0.15, 0.2, 0.5, 0.55)$.

We use $d_{1j} = d\left(x_{1j}^t, x_{NLC1}^{t+1}\right) = \left|x_{1j}^t - x_{NLC1}^{t+1}\right|$ and $d_{2j} = d\left(x_{2j}^t, x_{NLC2}^{t+1}\right) = p_j^{x_{NLC2}^{t+1}}\left(1-p_j\right)^{1-x_{NLC2}^{t+1}}$ as the similarity/dissimilarity function for the continuous and binary cluster-level variable. We use $\mathbf{h}\left(\mathbf{x}_j^t, \mathbf{x}_{NLC}^{t+1}\right) = \frac{1}{2}\left(\mathbf{x}_j^t + \mathbf{x}_{NLC}^{t+1}\right)$ as the function to integrate each $\mathbf{x}_{NLC}^{t+1}$ with the cluster-level variable $\mathbf{x}_j^t$ of the cluster that $\mathbf{x}_{NLC}^{t+1}$ joins. We set $n = 1,000$ for our simulation. The event probability $\boldsymbol{\eta}^{t+1}$ for the between-subject Multinomial response model for generating the multinomial response $\mathbf{z}^{t+1}$ in (1) is given by:

$$\eta_j^{t+1} = \frac{\exp\left(\beta_0 + \beta_1 d_{1j}^{t+1} + \beta_2 d_{2j}^{t+1}\right)}{1+\Sigma_{t+1}}, \quad \eta_{m_{(t+1)}}^{t+1} = \frac{1}{1+\Sigma_{t+1}},$$

$$\Sigma_{t+1} = \sum_{j=1}^{m_{(t+1)}-1} \exp\left(\beta_0 + \beta_1 d_{1j}^{t+1} + \beta_2 d_{2j}^{t+1}\right).$$

We set $\beta_0 = 1$, $\beta_1 = -0.15$ and $\beta_2 = 0.9$. We use a negative β_1 and positive β_2 so that smaller d_{1j} and larger d_{2j} will increase the probability of joining the

To reduce sampling variability as well as to compare estimates from fitting the between-subject multinomial response and independent logistic regression model, we controlled the total number of clusters to 10, i.e., $k_n = 10$, with the fixed sample size n. Thus, a newly infected case x_{NLC}^{t+1} will not form its own cluster for every t when $\sum_{j=1}^{k_t} z_j^{t+1} = 0$ until a certain number of samples are generated from $\text{Multi}_b\left(\boldsymbol{\eta}^{t+1}, 1\right)$ for a given number of clusters. For example, to grow the initial 5 clusters to 10 clusters, we select 6 subsample sizes, $1 < n_5 < n_6 \ldots < n_9 < n_{10}$, such that for $n_{l-1}+1 \le t \le n_l$ we generate $\mathbf{z}^{t+1}$ from $\text{Multi}_b\left(\boldsymbol{\eta}^{t+1}, 1\right)$ with k_t clusters, where $n_4 = 0$ and $5 \le l \le 10$. For our simulation study, we set $n_5 = 300, n_6 = 500, n_7 = 700, n_8 = 900, n_9 = 1100, n_{10} = 1500$.

We fit both the between-subject Multinomial response model and independent logistic regression as described in Section in (A.3). For the between-subject Multinomial response model, we obtained: $\widehat{\boldsymbol{\beta}} = \left(\widehat{\beta}_0, \widehat{\beta}_1\right)^\top = (-0.7995, 0.0999)^\top$, which were quite close to the respective true values $\beta_0 = -0.8$ and $\beta_1 = 0.1$. For the independent logistic regression, we obtained: $\widehat{\boldsymbol{\gamma}} = (\widehat{\gamma}_0, \widehat{\gamma}_1)^\top = (-2.0401, \widehat{\gamma}_1 = 0.0963)^\top$. Although $\widehat{\boldsymbol{\beta}}$ and $\widehat{\boldsymbol{\gamma}}$ are not directly comparable, we can compare the fitted $\widehat{\boldsymbol{\eta}}^{t+1}$ and $\widehat{\boldsymbol{\xi}}^{t+1}$ based on substituting $\widehat{\boldsymbol{\beta}}$ and $\widehat{\boldsymbol{\gamma}}$ in place of $\boldsymbol{\beta}$ and $\boldsymbol{\gamma}$. Since the independent logistic regression is fit to the subject vector $\mathbf{z}_{sub}^{t+1} = \left(z_1^{t+1}, z_2^{t+1}, \ldots, z_{k_t}^{t+1}\right)^\top$ of $\mathbf{z}^{t+1} = \left(z_1^{t+1}, z_2^{t+1}, \ldots, z_{k_t}^{t+1}, z_{k_t+1}^{t+1}\right)^\top$, we compare $\widehat{\boldsymbol{\xi}}^{t+1} = \left(\widehat{\xi}_1^{t+1}, \widehat{\xi}_2^{t+1}, \ldots, \widehat{\xi}_{k_t}^{t+1}\right)^\top$ with the normal-

ized subvector $\widehat{\boldsymbol{\eta}}_{sub}^{t+1} = \frac{1}{\widehat{s}_{t+1}}\left(\widehat{\eta}_1^{t+1}, \widehat{\eta}_1^{t+1}, \ldots, \widehat{\eta}_{k_t}^{t+1}\right)^\top$, where $\widehat{s}_{t+1} = \sum_{j=1}^{k_t} \widehat{\eta}_j^{t+1}$.

Shown below are the averaged $\widehat{\boldsymbol{\eta}}_{sub}^{t+1}$ and $\widehat{\boldsymbol{\xi}}^{t+1}$ over the samples $1 \le t \le n_5$ with 5 clusters, $\overline{\widehat{\boldsymbol{\eta}}}_{sub}^{5}$ and $\overline{\widehat{\boldsymbol{\xi}}}_{sub}^{5}$, and over the samples $n_9 \le t \le n_{10}$, $\overline{\widehat{\boldsymbol{\eta}}}_{sub}^{10}$ and $\overline{\widehat{\boldsymbol{\xi}}}_{sub}^{10}$:

$$\overline{\widehat{\boldsymbol{\eta}}}_{sub}^{5} = (0.1973, 0.1967, 0.2006, 0.2060, 0.1994)^\top,$$

$$\overline{\widehat{\boldsymbol{\xi}}}_{sub}^{5} = (0.2001, 0.1977, 0.2008, 0.2037, 0.1977)^\top,$$

$$\overline{\widehat{\boldsymbol{\eta}}}_{sub}^{10} = (0.1017, 0.1091, 0.1022, 0.0875, 0.1004, 0.1008, 0.1028, 0.0997, 0.0938, 0.1021)^\top,$$

$$\overline{\widehat{\boldsymbol{\xi}}}_{sub}^{10} = (0.1019, 0.1045, 0.1002, 0.0951, 0.1024, 0.0970, 0.0978, 0.1019, 0.0934, 0.1060)^\top.$$

In both cases, the averaged event probabilities from the fitted between-subject Multinomial response and independent logistic regression model were quite close to each other.

References 1. Wertheim JO, Kosakovsky Pond SL, Forgione LA, et al. Social and Genetic Networks of HIV-1 Transmission in New York City. PLoS Pathog. 2017;13(1):e1006000.

2. Novitsky V, Steingrimsson J, Howison M, et al. Longitudinal typing of molecular HIV clusters in a statewide epidemic. AIDS. 2021.

3. CDC. Respond. Ending the HIV Epidemic https://www.cdc.gov/endhiv/respond.html. Accessed April 4, 2021, 2021.

4. Wertheim JO, Panneer N, France AM, Saduvala N, Oster AM. Incident infection in high-priority HIV molecular transmission clusters in the United States. AIDS. 2020;34(8):1187-1193.

5. Oster AM, France AM, Panneer N, et al. Identifying Clusters of Recent and Rapid HIV Transmission Through Analysis of Molecular Surveillance Data. J Acquir Immune Defic Syndr. 2018;79(5):543-550.

6. Tumpney M, John B, Panneer N, et al. Human Immunodeficiency Virus (HIV) Outbreak Investigation Among Persons Who Inject Drugs in Massachusetts Enhanced by HIV Sequence Data. J Infect Dis. 2020;222(Suppl 5):S259-S267.

7. Amirkhanian Y, Kelly J, Kuznetsova A, et al. Using social network methods to reach out-of-care or ART-nonadherent HIV+ injection drug users in Russia: addressing a gap in the treatment cascade. J Int AIDS Soc. 2014;17(4 Suppl 3):19594.

8. Kobe J, Talbot O, Chen I, et al. Short Communication: Viral Genetic Linkage Analysis Among Black Men Who Have Sex With Men (HIV Prevention Trials Network 061). AIDS Res Hum Retroviruses. 2019;35(5):434-436.

9. Janulis P, Phillips G, Birkett M, Mustanski B. Sexual Networks of Racially Diverse Young MSM Differ in Racial Homophily But Not Concurrency. J Acquir Immune Defic Syndr. 2018;77(5):459-466.

10. Aitken CK, Higgs P, Bowden S. Differences in the social networks of ethnic Vietnamese and non-Vietnamese injecting drug users and their implications for blood-borne virus transmission. Epidemiol Infect. 2008;136(3):410-416.

11. Wu Z, Detels R, Zhang J, et al. Risk factors for intravenous drug use and sharing equipment among young male drug users in Longchuan County, south-west China. AIDS. 1996;10(9):1017-1024.

12. Cyrus E, Clarke R, Hadley D, et al. The Impact of COVID-19 on African American Communities in the United States. Health Equity. 2020;4(1):476-483.

13. Furuse Y, Sando E, Tsuchiya N, et al. Clusters of Coronavirus Disease in Communities, Japan, January-April 2020. Emerg Infect Dis. 2020;26(9).

14. Andalibi A, Koizumi N, Li MH, Siddique AB. Symptom and Age Homophilies in SARS-CoV-2 Transmission Networks during the Early Phase of the Pandemic in Japan. Biology (Basel). 2021;10(6).

15. Kenyon C, Colebunders R. Birds of a feather: homophily and sexual network structure in sub-Saharan Africa. Int J STD AIDS. 2013;24(3):211-215.

16. Voeten H, Sikkema RS, Damen M, et al. Unravelling the modes of transmission of SARS-CoV-2 during a nursing home outbreak: looking beyond the church super-spread event. Clin Infect Dis. 2020.

17. Danis K, Epaulard O, Benet T, et al. Cluster of Coronavirus Disease 2019 (COVID-19) in the French Alps, February 2020. Clin Infect Dis. 2020;71(15):825-832.

18. Little SJ, Chen T, Wang R, et al. Effective HIV Molecular Surveillance Requires Identification of Incident Cases of Infection. Clin Infect Dis. 2021.

19. Little, S. J., Kosakovsky Pond, S. L., Anderson, C. M., Young, J. A., Wertheim, J. O., Mehta, S. R., . . . Smith, D. M. (2014). Using HIV networks to inform real time prevention interventions. PLoS ONE, 9(6), e98443. doi:10.1371/journal.pone.0098443

Acknowledgements: NIH R24 AI106039-05, NIH R37 AI51164 (DeGruttola), NIH CFAR AI036214.
